# Different Expression Characteristics of LAG3 and PD-1 in Sepsis and Their Synergistic Effect on T Cell Exhaustion: A New Strategy for Immune Checkpoint Blockade

**DOI:** 10.3389/fimmu.2019.01888

**Published:** 2019-08-07

**Authors:** Bailin Niu, Fachun Zhou, Yanxin Su, Long Wang, Yuanyuan Xu, Ziying Yi, Yushen Wu, Huimin Du, Guosheng Ren

**Affiliations:** ^1^Department of Emergency, The First Affiliated Hospital of Chongqing Medical University, Chongqing, China; ^2^Department of Intensive Care Medicine, The First Affiliated Hospital of Chongqing Medical University, Chongqing, China; ^3^Chongqing Key Laboratory of Molecular Oncology and Epigenetics, The First Affiliated Hospital of Chongqing Medical University, Chongqing, China; ^4^Department of Oncology, The First Affiliated Hospital of Chongqing Medical University, Chongqing, China; ^5^Department of Endocrine and Breast Surgery, The First Affiliated Hospital of Chongqing Medical University, Chongqing, China

**Keywords:** T cell exhaustion, sepsis, PD-1, LAG3, synergistic inhibition

## Abstract

The impairment of immunity characterized by T cell exhaustion is the main cause of death in patients with sepsis after the acute phase. Although PD-1 blockade is highly touted as a promising treatment for improving prognosis, the role of PD-1 plays in sepsis and particularly its different roles in different periods are still very limited. A recent study revealed LAG3 can resist the therapeutic effect of PD-1 blockade in tumor, which inspired us to understand their role in sepsis. We enrolled 26 patients with acute sepsis from 422 candidates using strict inclusion criteria. Follow-up analysis revealed that the expression levels of PD-1 were rapidly increased in the early stage of sepsis but did not change significantly as infection continued (*P* < 0.05). However, the expression of LAG3 was contrary to that of PD-1. Compared with LAG3 or PD-1 single-positive T cells, T cells coexpressing LAG3 and PD-1 were significantly exhausted (*P* < 0.05). The proportion of coexpressing T cells was negatively correlated with the total number of lymphocytes (*r* = −0.653, *P* = 0.0003) and positively correlated with the SOFA score (*r* = 0.712, *P* < 0.0001). In addition, the higher the proportion of coexpressing T cells was, the longer the hospital stay and the higher the mortality. These results showed that LAG3 and PD-1 had a potential synergistic effect in regulating the gradual exhaustion of T cells in sepsis, which seriously affected the clinical prognosis of patients. Therefore, LAG3 and PD-1 double-positive T cells are an important indicator for immunity detection and prognostic evaluation. In the future, precision therapy may pay more attention to the different expression patterns of these two molecules.

## Highlights

- In sepsis, LAG3 and PD-1 had unique expression characteristics in T cells, and the T cells that coexpress LAG3 and PD-1 were significantly exhausted.
- The proportion of T cells coexpressing LAG3 and PD-1 was negatively correlated with the total number of lymphocytes and positively correlated with the SOFA score.
- In septic patients, the higher the proportion of LAG3 and PD-1-coexpressing T cells was, the longer the hospital stay and the higher the mortality.

## Introduction

Sepsis is characterized by an intense systemic response to infection. The incidence rate is estimated to be up to 30 million cases and 6 million deaths worldwide per year, and the number of cases is rising (1) and has become the leading cause of death in intensive care units (2, 3). The pathogenesis of sepsis is the result of a complex network of events involving proinflammatory and anti-inflammatory processes triggered by the infectious agent (4). Postmortem studies of patients who died of sepsis have provided important insights into why septic patients die and highlighted key immunological defects that impair host immunity (5, 6). One of the most important features of immunosuppression is T cell exhaustion (7, 8). Many factors are involved in this process, and negative costimulatory molecules are considered to be the very important elements (5, 8–11). Recently, some negative costimulatory molecules have shown interactive relationships in non-septic disease, and these relationships seriously affect the occurrence and development of disease, particularly the relationship between lymphocyte-activation gene 3 (LAG3) and programmed cell death 1 (PD-1) (12–15). In particular, a recent study showed that the activation of LAG3 can resist the efficacy of anti-PD-1/B7-H1 therapy in tumor (16), and dual blockade of LAG3 and PD-1 can provoke more powerful antitumor or antiviral effects than the sum of blocking each molecule alone (12, 13, 15, 17–20). However, whether they also interact in sepsis which is different from the chronic pathological changes mentioned above has not been studied. Here, we performed a prospective observational study and systematically analyzed the expression characteristics and functions of LAG3 and PD-1 in T cells as well as the relationship between LAG3 and PD-1 and the prognosis of patients with sepsis.

## Materials and Methods

### Patients' Enrollment and Clinical Data Collection

**Figure 1:**
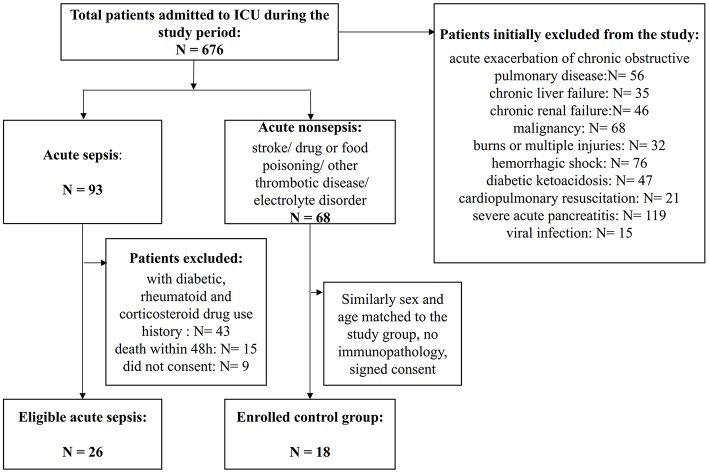
Flowchart of patient enrollment. A total of 676 patients were admitted to the ICU during the study period, and among them, 422 patients were identified as having severe infection. However, many patients had associated chronic immunocompromising diseases or drug use or acute exacerbations of chronic infections and needed to be excluded. Finally, 26 patients with sepsis were enrolled, and 18 patients with similar critical scores that were sex- and age-matched non-septic patients were enrolled as the controls.

Adult patients with suspected infection from the emergency department admitted to the medical and surgical ICU at the First Affiliated Hospital of Chongqing Medical University were followed up and screened for sepsis daily, using the sepsis 3.0 criteria (21), and the organ damage was assessed via the sequential [sepsis-related] organ failure assessment (SOFA) score (22). All patients needed to meet the criteria of sepsis 3.0 upon enrollment. Patients with end-stage chronic diseases, such as uremia and liver failure, active malignancy, death within 48 h, or chronic viral infection, such as HIV, hepatitis B or C; taking immunosuppressive medications with corticosteroids at doses ≥10 mg prednisone or equivalent per day (23); or diagnosed with other diseases that could also affect host immunity, as shown in Figure 1, were excluded. The control subjects consisted of age- and sex-matched non-septic critical patients with the same APACHE II scores to study group, and the main diseases were stoke, myocardial infarction or acute intoxication, and none of them had any of the immunocompromised diseases mentioned above. Details of the sepsis patient and control subjects are shown in Figure 1 and Table 1. Other relevant clinical data were also collected, mainly including average hospital stay, mortality rate, absolute number of peripheral blood lymphocytes and SOFA score related indicators. Informed consent is required and obtained from the legally authorized patient representatives, due to all patients admitted to the ICU were judged to be too seriously ill to provide valid consent.

**Table 1:** Clinical characteristics of patients with sepsis and controls.

**Parameter**	**Patients with sepsis (*n* = 26)**	**Control subjects (*n* = 18)**	***P*-value**
Males, percentage (number)	61.5 (16)	61.1 (11)	0.382
Age, years (range)	50 (32 to 78)	53 (38 to 82)	0.057
SOFA score at admission, median (range)	7.6 (3 to 16)	5.9 (3 to 13)	0.055
APACHE II score at admission, median (range)	16.5 (12 to 32)	14.7 (8 to 24)	0.062
28-day mortality, number (percentage)	5 (19.23)	4 (22.22)	0.217
Ventilation days, median days (range)	5.5 (2 to 17)	9.6 (2 to 26)	<0.001
Length of ICU stay, median days (range)	7.5 (3 to 22)	9.2 (4 to 32)	0.052
Length of hospitalization, median days (range)	16.4 (7 to 36)	25.6 (6 to 89)	0.008
White blood cell at intake, mean (× 10^9^/L) (range)	13.66 (2.8 to 38.5)	10.12 (5.8 to 14.5)	0.032
Absolute lymphocyte count, median (× 10^9^/L) (range)	0.71 (0.28 to 1.22)	1.42 (0.98 to 2.31)	<0.001
Procalcitonin, median (nanogram /L) (range)	45.5 (10 to 286)	0.32 (0.05 to 1.32)	<0.001
C-reactive protein, median (milligram /L) (range)	82.4 (42 to 142)	47.5 (22 to 92)	<0.001
Shock^*^, number (percentage)	20 (76.9)	1 (5.56)	<0.001
Biliary tract infection, number (percentage)	6 (23.1)	n/a	n/a
Urinary system infection, number (percentage)	9 (34.6)	n/a	n/a
Pelvic and abdominal cavity infection, number (percentage)	4 (15.4)	n/a	n/a
Other site infection, number (percentage)	7 (26.9)	n/a	n/a

APACHE II, Acute Physiology and Chronic Health Evaluation II; SOFA, Sequential [Sepsis-related] Organ Failure Assessment; n/a, Not Applicable; Shock^*^, the patients with septic shock can be clinically identified by a vasopressor requirement to maintain a mean arterial pressure of 65 mm Hg or greater and serum lactate level >2 mmol/L (> 18 mg/dL) in the absence of hypovolemia (24).

### Sample Collection and Primary Treatment

About 5 ml blood with disodium ethylenediaminetetraacetate dihydrate (EDTA-2Na) anticoagulation was collected through an indwelling central venous catheter or venipuncture on day 1 and again on day 5. The blood was instantly processed in our laboratory. Peripheral blood mononuclear cells (PBMCs) were isolated via Ficoll-Hypaque density gradient centrifugation following standard protocols. The cells were washed and resuspended in T cell medium (Roswell Park Memorial Institute (RPMI) 1640 medium supplemented with 10% heat-inactivated fetal bovine serum (FBS), penicillin and streptomycin at an active concentration of 100 units per milliliter each, L-glutamine and non-essential amino acids) and processed for flow cytometry, proliferation or cytokine secretion evaluations as described below.

### Antibodies and Reagents

All flow cytometry antibodies were purchased from BD Pharmingen (San Diego, CA, USA), BioLegend (San Diego, CA, USA) and KeyGen Biotech (Nanjin, Jiangsu, China). The following antibodies were obtained from BD Pharmingen™: CD3-APC-Cy™7, CD4-FITC, CD8-PECy™5, PD-1-APC, and LAG3-PE. The following reagents were obtained from BioLegend: PerCP/Cy5.5-conjugated anti-IL-2, PE/Cy7-conjugated anti-IL-6, PerCP/Cy5.5-conjugated anti-TNF-α, and PE/Cy7-conjugated anti-IFN-γ antibodies, a FITC Annexin V/PI kit, and a KGA: FITC-BrdU kit. The following quantum MESF beads were purchased from Bangs Laboratories: Fluorescent Microspheres, Intensity Standard: Dragon Green, Flash Red, PE-MESF, and APC-MESF. The PMA/ionomycin mixture (250X) was purchased from MultiSciences (Lianke) Biotech (Hangzhou, Zhejiang, China). Enzyme-linked immuno sorbent assay (ELISA) kits for human interleukin-2 (IL-2), IL-6, tumor necrosis factor-alpha (TNF-α) and interferon-gamma (IFN-γ) were purchased from Beijing 4A Biotech (Beijing, China). Brefeldin A was purchased from Qcbio Science & Technologies Co., Ltd. (Beijing, China).

### Flow Cytometry, Immunofluorescence, Proliferation, and Cytokine Analysis

Target cells were collected at various time points and stained with the appropriate antibodies. The samples were run on a FACS flow cytometer (BD Biosciences) and analyzed by FCS Express. For surface marker staining, 100 μl of PBMCs was incubated with 20 μl of human AB serum and the indicated fluorescently conjugated antibodies for 1 h at room temperature. The expression levels of LAG3 and PD-1 were observed by laser-focused microscopy. Then, the cells were extensively washed in PBS with 1% bovine serum albumin (BSA) and resuspended in PBS with 2% BSA and 2% paraformaldehyde (PFA). Viable lymphocytes were identified and gated by forward scatter (FSC) and side scatter (SSC) properties. T cells were identified as CD3^+^, with subtypes of CD4^+^ T and CD8^+^ T cells. For proliferative and cytokine secretion function detection, PD-1^−^LAG3^−^, PD-1^+^LAG3^−^, PD-1^−^LAG3^+^, and PD-1^+^LAG3^+^ cells were sorted to more than 90% purity by FACS, labeled with CFSE (2 μM) for 10 min, cultured in 24-well plates, activated by α-CD3/α-CD28, and stimulated with PMA (50 ng/ml) and ionomycin (500 ng/ml) for 48 h. The culture supernatant was harvested at 12 and 48 h following stimulation, and cytokine levels were determined using ELISA kits according to the manufacturer's instructions. We measured human IL-2 and IL-6 levels for CD4^+^ T cells and TNF-α and IFN-γ levels for CD8^+^ T cells. Furthermore, we also determined the amounts of intracellular cytokines that were synthesized but not secreted via flow cytometry. Briefly, after the stimulation described above, Brefeldin A, an intracellular protein transport inhibitor, was added into the culture system. Forty-eight hours later, the T cells were collected, fixed with 4% paraformaldehyde, treated with 0.1% Triton-100 and flow cytometry antibodies, and then analyzed by flow cytometry.

### Laboratory Fluorescence Quantitation

In order to quantitatively detect the expression level of related receptors, quantum MESF beads were run with each flow cytometric assay. The Quantum beads are microspheres that each has a fixed fluorescence. The corresponding fluorescence peaks were obtained when the microspheres were run on a flow cytometer to provide individual peaks (six peaks for FITC and five peaks for PE). These peaks of known fluorescence intensities were converted to Molecules of Equivalent Soluble Fluorochrome (MESF) units using QuickCal™ software to generate a standard curve. The mean fluorescence intensity (MFI) of each marker was converted to MESF units based on the Quantum Bead MESF standard curve, according to a previously described method (25, 26).

### Statistical Analysis

Data were analyzed with the statistical software Prism version 7 (GraphPad, San Diego, CA, USA), and expressed as the mean ± SEM or shown as a box plot. For comparisons of two groups, Student's *t*-test was employed. One-way ANOVA with Tukey's multiple comparison test was used to analyze data containing more than two groups. For survival studies, a log-rank test was used. Two-tailed non-parametric Wilcoxon matched pairs test, two-tailed Mann-Whitney U test and the Kruskal-Wallis test were used for non-parametric data. To test for correlations, Pearson's simple correlation coefficient was applied. *P* < 0.05 were considered to indicate statistically significant differences.

## Results

### Patient Enrollment and Specimen and Clinical Data Collection

A flowchart of patient enrollment is shown in Figure 1. Ultimately, 26 subjects with acute sepsis and 18 control subjects were included in the study. The clinical characteristics of the patients with sepsis and controls are displayed in Table 1. There were no significant differences in gender, age, SOFA scores and APACHE II scores, 28-day mortality, and length of ICU stay between the two groups, and the *P*-values were 0.382, 0.057, 0.55, 0.062, 0.217, and 0.052, respectively. However, the differences in the WBC, ventilation days, and length of hospitalization, procalcitonin, C–reactive protein, and the percentage of shock patient between the two groups were conspicuous (*P* < 0.05). The absolute number of lymphocytes decreased more significantly in the sepsis group (*P* < 0.001).

### Expression Characteristics of PD-1 and LAG3 in Peripheral T Cells During the Onset of Sepsis

**Figure 2:**
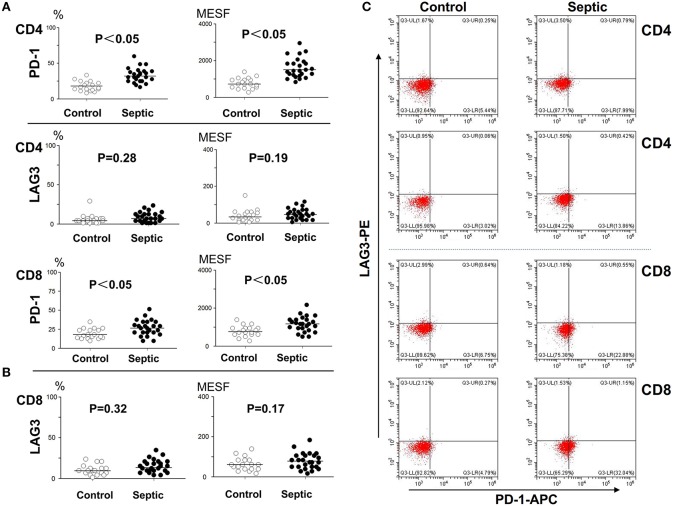
Determination of the expression of LAG3 and PD-1 on CD4 and CD8 T cells at the onset of sepsis. The peripheral blood of septic patients and controls were collected within 12 h after enrollment, and then stained for flow cytometry. The positive rate relative to that of the isotype control staining was determined. Fluorescence intensity was quantified by calculating MESF values as described in the procedures. The horizontal lines represent the mean levels. Scattergram **(A)** and **(B)** showed the expression of LAG3 and PD-1 on CD4 and CD8 Tcells, respectively. Representative flow cytometry pictures **(C)**. *P*-values were calculated with a non-parametric 2-tailed Mann-Whitney U test.

The patients admitted to hospital with infection were screened daily for sepsis, and the moment when they reached the sepsis criteria was identified as onset of sepsis. To determine the number of lymphocytes and the expression of LAG3 and PD-1 on CD4^+^ T and CD8^+^ T lymphocyte surfaces, blood was obtained within 12 h of the onset of sepsis and extensively characterized by flow cytometry. An identical analysis was performed on the non-septic control subjects to provide comparative data. In the patients in the acute phase of sepsis, the expression of PD-1 on both CD4^+^ T cells and CD8^+^ T cells was significantly elevated compared with that in the controls (*P* < 0.05) (Figure 2). However, the expression of LAG3 on CD4^+^ T cells or CD8^+^ T cells was not obviously elevated in the sepsis group compared with the control group, and the *P*-values were 0.28 and 0.19 for the expression rate (%) and expression intensity (MESF) of CD4^+^ T cells, respectively, and 0.32 and 0.17 for the expression rate (%) and MESF of CD8^+^ T cells, respectively (Figure 2).

### Changes in PD-1 and LAG3 Expression Over the Course of Acute Sepsis

**Figure 3:**
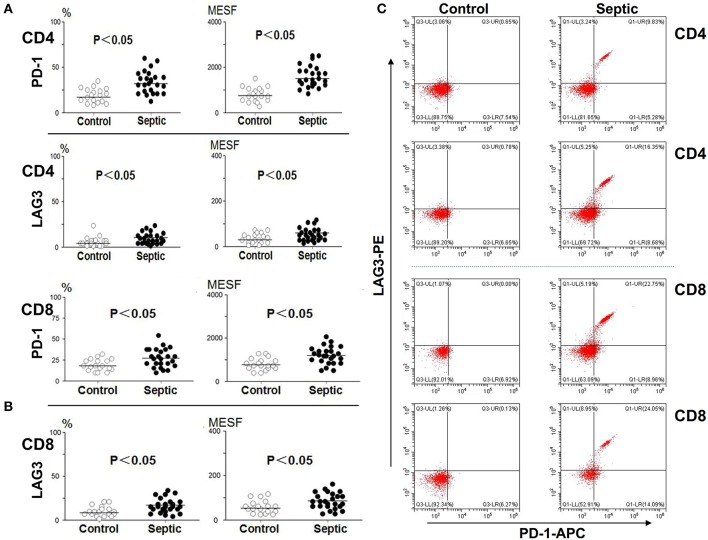
The expression of LAG3 and PD-1 on CD4 and CD8 T on the 5th day of the sepsis. The peripheral blood of septic patients and controls were collected at enrollment and again 5 days later, and analyzed by flow cytometry for the expression of the indicated markers on CD4 T cells and CD8 T cells. The positive proportion and MESF values were obtained as described in the procedures. The horizontal lines represent the mean levels. Scattergram **(A)** and **(B)** showed the expression of LAG3 and PD-1 on CD4 and CD8 Tcells, respectively. Representative flow cytometry pictures **(C)**. *P*-values were calculated with a non-parametric 2-tailed Mann-Whitney U test.

**Figure 4:**
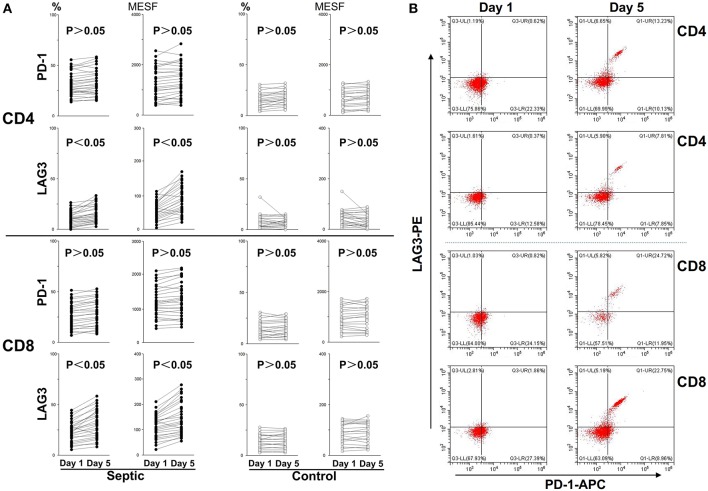
Changes in LAG3 and PD-1 expression on CD4 and CD8 T cells over the course of acute sepsis. The lines connect values at enrollment and the 5th day after the onset of sepsis or on the 5th day in the control group. The positive proportion and MESF values were obtained as described in the procedures. Scattergram **(A)** showed the expression changes of LAG3 and PD-1 on CD4 and CD8 Tcells, respectively. Representative flow cytometry pictures of sepsis group from day 1 to day 5 **(B)**. As there were no significant changes in the control group from day 1 to day 5, we did not present representative images. *P*-values were calculated with a 2-tailed, non-parametric Wilcoxon matched pairs test.

Blood was collected from the patients and controls on the 5th day and analyzed. As presented in Figure 3, compared with the control group, the sepsis group exhibited obviously higher PD-1 expression levels on both CD4^+^ and CD8^+^ T cells (*P* < 0.05). In addition, the LAG3 expression levels were also distinctly elevated on both CD4^+^ and CD8^+^ T cells (*P* < 0.05) (Figures 3A,B). We separately compared the changes in PD-1 and LAG3 expression on CD4^+^ and CD8^+^ T cells in the septic patients. Interestingly, there were no significant differences in the expression of PD-1 on CD4^+^ or CD8^+^ T cells between the day of onset and the 5th day of sepsis (*P* > 0.05) (Figures 4A,B). Nonetheless, LAG3 had a trend toward an increase in the expression rate (%) (*P* < 0.05) and per cell intensity (MESF) (*P* < 0.05) from day 1 to day 5 in both CD4^+^ and CD8^+^ T cells (Figures 4A,B).

### Co-expression of PD-1 and LAG3 on T Cells in the Extended Phase of Sepsis

**Figure 5:**
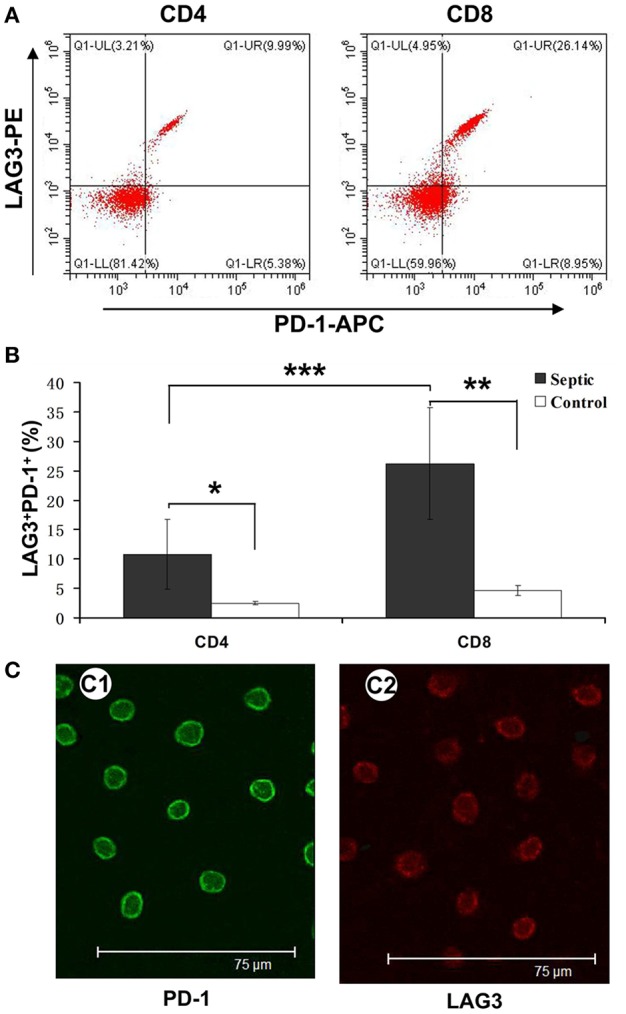
Determination of the coexpression of LAG3 and PD-1 on CD4 and CD8 T cells over the course of acute sepsis. Peripheral blood was collected at enrollment and again 5 days later from patients with sepsis and normal controls. We could directly observe T cells with high expression of LAG3 and PD-1 by immunofluorescence **(C)**. After analysis by flow cytometry for the expression of the indicated markers on CD4- and CD8-positive T cells, the ratio of CD8^+^ T cells with LAG3 and PD-1 coexpression was close to 25% and higher than that of CD4^+^ T cells, which was approximately 8% **(A)**; ***P < 0.05 CD4^+^ T cells vs. CD8^+^ T cells in sepsis **(B)**. However, both ratios were significantly higher than those for the control group; *P < 0.05 and **P < 0.05, respectively **(B)**. Data are presented as mean ± SEM, Student's *t*-test was employed.

As mentioned above, we analyzed the negative costimulatory molecules PD-1 and LAG3 to determine the expression and changes in these molecules in both CD4^+^ and CD8^+^ T cells. Here, we further analyzed their coexpression on the same CD4^+^ and CD8^+^ T cells collected on the 5th day of sepsis. The PD-1 and LAG3 coexpression rates were significantly elevated for both the CD4^+^ and CD8^+^ T cells from the septic patients compared with those from the controls (^*^P < 0.05;^**^P < 0.05) (Figure 5A); however, the proportion of coexpressing CD8^+^ T cells was significantly higher than that of coexpressing CD4^+^ T cells (^***^P < 0.05) (Figure 5B). By immunofluorescence, the expression of LAG3 and PD-1 on the surface of T cells on the 5th day of sepsis was also directly observed (Figure 5C).

### Stimulated T Cells With Different Phenotypes of Cytokine Secretion, Proliferation, and Apoptosis in Patients With Sepsis

**Figure 6:**
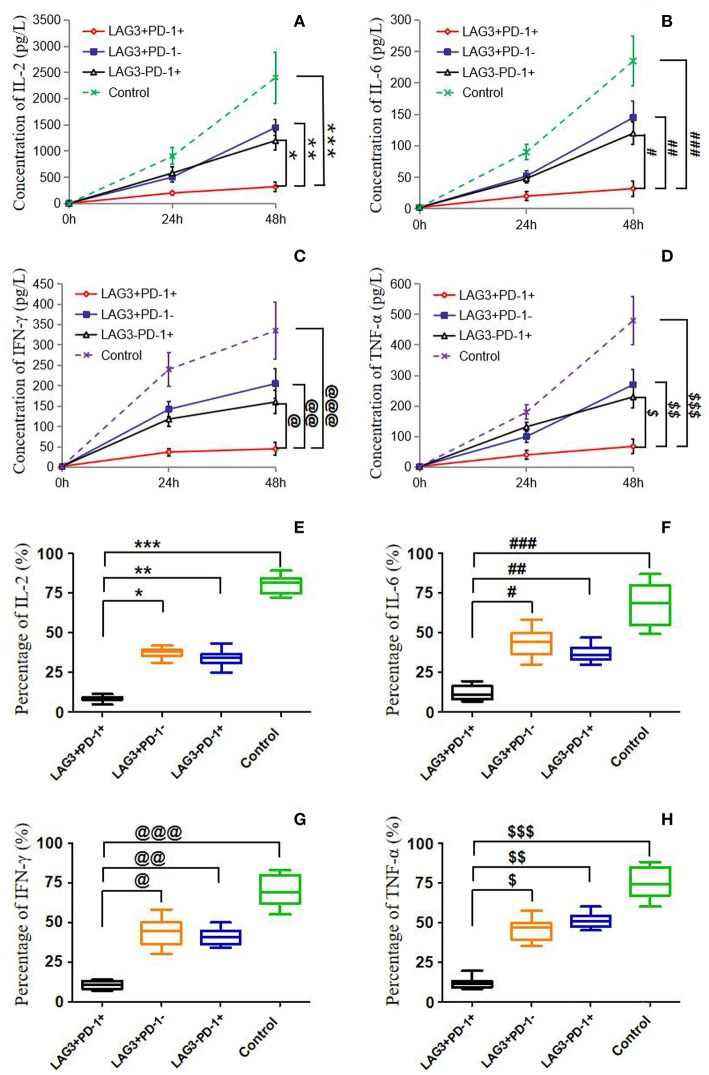
Determination of the cytokine secretion by T cells with single- or double-positive expression of LAG3 and PD-1. As described before, T cells with different phenotypes were sorted via FACS, cultured in 24-well plates, activated by α-CD3/α-CD28 and stimulated with PMA (50 ng/ml) and ionomycin (500 ng/ml) for 48 h. Cytokine concentrations in the supernatant were determined by ELISA **(A–D)**, and intracellular cytokine levels were measured by flow cytometry **(E–H)**. IL-2 **(A,E)** and IL-6 **(B,F)** were measured mainly to evaluate the function of CD4^+^ T cells, and IFN-γ **(C,G)**, and TNF-α **(D,H)** were measured mainly to detect the function of CD8^+^ T cells. There was a common trend in cytokine secretion capacity, that is, the function of LAG3 and PD-1-coexpressing T cells was significantly weaker than that of LAG3 or PD-1 single-positive T cells and control T cells; *P < 0.05, **P < 0.05,***P < 0.05; ^@^*P* < 0.05, ^@@^*P* < 0.05, ^@*@@*^*P* < 0.05; ^#^*P* < 0.05, ^*###*^*P* < 0.05, ^*###*^*P* < 0.05; ^*$*^*P* < 0.05, ^*$$*^*P* < 0.05, and ^*$$$*^*P* < 0.05. In particular, the T cells coexpressing LAG3 and PD-1 exhibited decreased cytokine secretion that was more than 2-fold lower than the secretion of the single-positive T cells. Data of **(A–D)** are expressed as the mean ± SEM, and One-way ANOVA with Tukey's multiple comparison test was used. Data of **(E–H)** are shown as a box plot and analyzed using the Kruskal-Wallis Test due to variance inhomogeneity.

**Figure 7:**
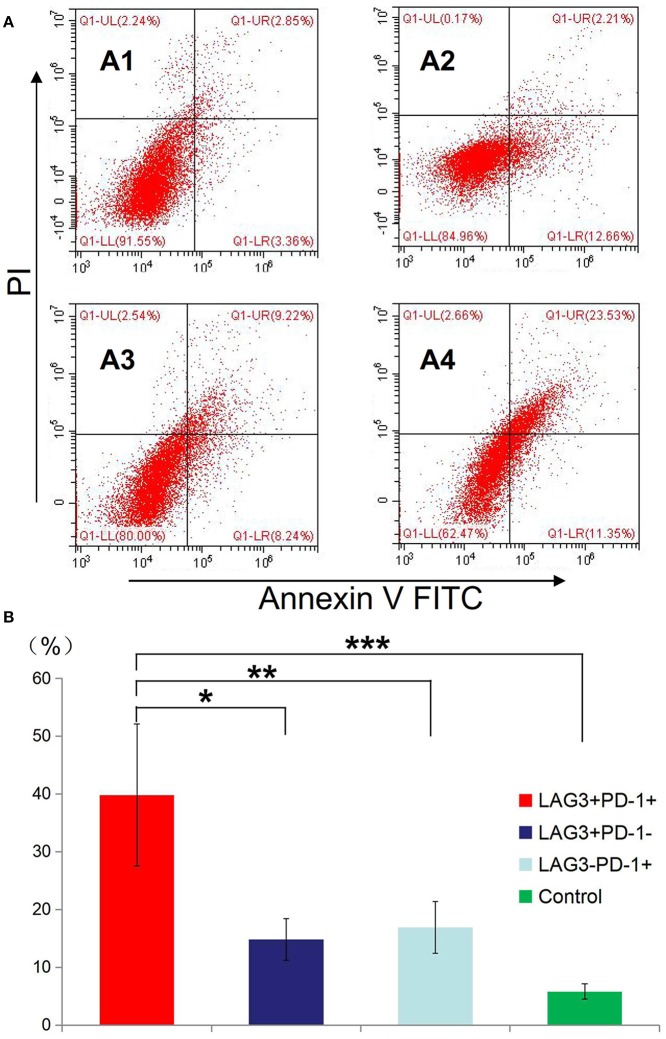
Determination of the apoptosis rates of T cells with double- or single-positive expression of LAG3 and PD-1. T cells from the peripheral blood of subjects and controls were sorted and cultured for 48 h. The apoptosis rates of T cells with different phenotypes including the T cells from controls **(A1)**, LAG3^+^PD-1^−^ T cells **(A2)**, LAG3^−^PD-1^+^ T cells **(A3)**, and LAG3^+^PD-1^+^ T cells **(A4)** were determined by flow cytometry. We found that the apoptosis rates of the LAG3^+^PD-1^+^ T cells were significantly higher than those of the LAG3^+^PD-1^−^ T cells (*P < 0.05), LAG3^−^PD-1^+^ T cells (**P < 0.05), and control T cells (***P < 0.05) **(B)**. The early and late apoptosis rates of the LAG3^+^PD-1^+^ T cells were far >2-fold higher than those of the LAG3^+^PD-1^−^ T cells and LAG3^−^PD-1^+^ T cells. Data are expressed as the mean ± SEM, and One-way ANOVA with Tukey's multiple comparison test was used.

**Figure 8:**
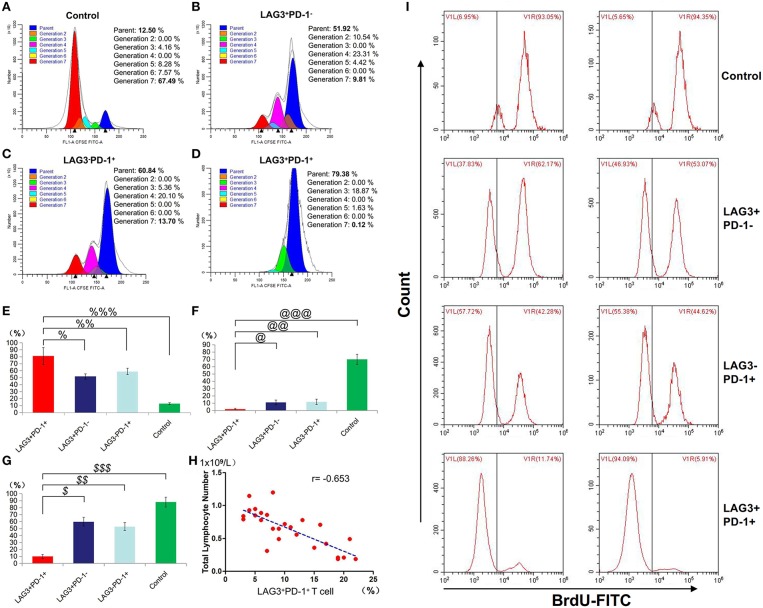
Determination of the proliferation of T cells with double- or single-positive expression of LAG3 and PD-1. After the sorting and culturing described above, we used two methods, CFSE **(A–F)** and FITC-Brdu **(G)** staining, to consistently evaluate the proliferative capacities of T cells with different phenotypes. From the division and proportional analysis, we found that the proliferation of LAG3^+^PD-1^+^ T cells was obviously inhibited **(A)** compared with that of LAG3^+^PD-1^−^ T cells **(B)**, LAG3^−^PD-1^+^ T cells **(C)**, and control T cells **(D)**, with the highest proportion of cells remaining in the parental generation (^%^*P* < 0.05, ^*%%*^*P* < 0.05, and ^*%%%*^*P* < 0.05) **(E)** and the lowest proportion of cells appearing in the seventh generation (^@^*P* < 0.05, ^@@^*P* < 0.05, and ^@*@@*^*P* < 0.05) **(F)**. The analysis of FITC-Brdu staining showed similar results, with the proliferation rates of LAG3 and PD-1 double-positive T cells being the lowest (^*$*^*P* < 0.05, ^*$$*^*P* < 0.05, and ^*$$$*^*P* < 0.05) **(G)**. The proportion of coexpressing T cells was negatively correlated with the total number of lymphocytes using Pearson's simple correlation coefficient (*r* = −0.653, 95%CI: −0.831 to −0.356, *P* = 0.0003) **(H)**. Data of **(E–G)** are expressed as the mean ± SEM, and compared with ANOVA. Representative pictures of flow cytometry using BrdU-FITC to detect the proliferation rates of T cells with different phenotypes **(I)**.

We conducted cell sorting for cells with different phenotypes in the peripheral blood of the patients with sepsis and controls on the 5th day after enrollment through flow cytometry. After standardizing the concentrations, the different types of T cells including PD-1^+^LAG3^−^ T cells, PD-1^−^LAG3^+^ T cells, PD-1^+^LAG3^+^ T cells and control T cells were cultured in 24-well plates at the same concentration and then activated and stimulated for 48 h. We found that the levels of IL-2 and IL-6 mainly secreted by CD4^+^ T cells and IFN-γ and TNF-α mainly secreted by CD8^+^ T cells were lowest in PD-1^+^LAG3^+^ T cells groups (Figures 6A–D), and regardless of the terminal concentration or the rate of secretion increase, the levels of these factors were all significantly lower in the PD-1^+^LAG3^+^ T cell group than in the other three groups. We also determined the intracellular levels of these cytokines in the different groups. The intracellular cytokine levels and extracellular supernatant cytokine concentrations showed the same trends, as displayed in Figures 6E–H. Moreover, the levels of these cytokines were extremely low in the PD-1^+^LAG3^+^ T cell groups compared with the other groups. Based on the detection of apoptosis, we found that the total (early and late) apoptosis rates were most increased in the PD-1^+^LAG3^+^ T cell groups compared with the other groups (Figure 7B). As for proliferative function, we used CFSE staining for cell division analysis (Figures 8A–G) and FITC-BrdU staining for proliferation rate determination (Figure 8G). After activation and stimulation, the T cells of the control groups rapidly divided and proliferated; only approximately 12% of the parental cells could be detected, and the seventh generation cells accounted for approximately 65% (Figure 8A). In contrast, the PD-1^+^LAG3^+^ T cells showed very slow division and proliferation rates, with more than 80% of the parental cells not undergoing division or proliferation, and seventh generation cells were barely detectable (Figure 8D). Although the proliferative functions of the PD-1^−^LAG3^+^ T cells and PD-1^+^LAG3^−^ T cells were damaged, their proliferative capacities were still significantly higher than those of the PD-1^+^LAG3^+^T cells (Figures 8A–G). Furthermore, we also found that the absolute number of lymphocytes in the patients with sepsis was negatively correlated with the proportion of LAG3 and PD-1 double-positive T cells (*r* = −0.653, 95%CI: −0.831 to −0.356, *P* = 0.0003) (Figure 8H).

### Statistical Analysis of Other Clinical Data: The SOFA Score, Hospitalization Days and Mortality

**Figure 9:**
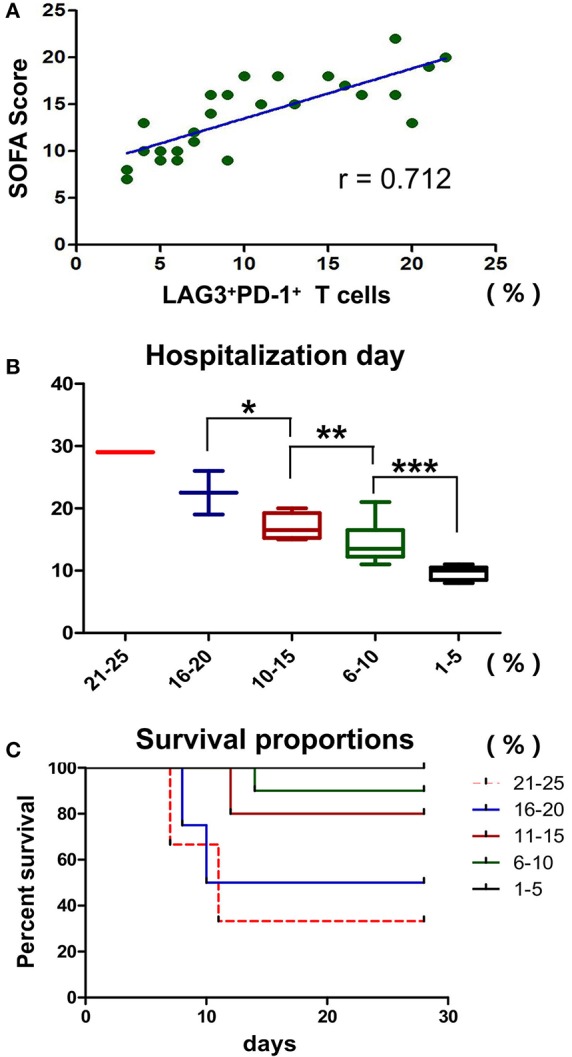
Effects of T cells with LAG3 and PD-1 double-positive expression on the clinical indicators and prognosis of patients with sepsis. The proportion of coexpressing T cells was positively correlated with the SOFA score using Pearson's simple correlation coefficient (*r* = 0.712, 95%CI: 0.448 to 0.862, *P* < 0.0001) **(A)**. Due to the small number of patients, we compared the proportions of LAG3 and PD-1-coexpressing T cells by subgroups in the mortality and survival analyses, and we set 5% as the group increment. The numbers of patients with 1–5%, 6–10%, 11–15%, 16–20%, and 21–25% double-positive T cells were 5, 9, 5, 4 and 3, respectively. The higher the proportion of coexpressing T cells was, the longer the hospital stay (*P < 0.05, **P < 0.05, and ***P < 0.05) **(B)**, analyzed by Kruskal-Wallis Test. For the survival analysis, a log-rank test was performed. We found that there were differences in survival among the high-proportion subgroups (*P* < 0.05, a vs. b; *P* < 0.05, a vs. c; *P* < 0.05, b vs. c; *P* < 0.05, a vs. d; and *P* < 0.05, a vs. e) and no differences among the low-proportion subgroups (*P* > 0.05, e vs. d and *P* > 0.05, d vs. c) **(C)**.

Previously, we showed that LAG3 and PD-1 double-positive T cells are significantly impaired in terms of proliferation and antiapoptosis function and that the higher the proportion of double-positive T cells is, the lower the absolute number of lymphocytes in patients. Do these cells have any influence on other clinical indicators in patients with sepsis? Here, the SOFA score, hospitalization days and mortality were analyzed. We found that the proportion of LAG3 and PD-1 double positive T cells was positively correlated with the SOFA score (*r* = 0.712, 95%CI: 0.448 to 0.862, *P* < 0.0001) (Figure 9A). To understand the relationship between the proportion of LAG3 and PD-1 double-positive T cells and the length of hospital stay, mortality or overall survival, we stratified the proportions at 5% intervals including 1–5%, 6–10%, 11–15%, 16–20%, 21–25%, and no subject had a proportion above 25%. Figure 9B shows that the higher the double-positive proportion was, the longer the hospital stay, and there were significant differences among the proportion ranges. Similar trends were observed for mortality and overall survival, specifically, the higher the proportion range was, the higher the death rate and lower the survival (Figure 9C).

## Discussion

Sepsis, a systemic inflammatory condition due to severe infection, has become the most common cause of mortality in most intensive care units (27–29). Improved treatment protocols and updated organ support equipment have resulted in the majority of patients surviving the initial 72 h of sepsis only to succumb later in the time course of the disease (30, 31). The failure of several high-profile clinical trials in sepsis has led basic and clinical researchers to state that sepsis studies need a new direction, and there is increasing recognition that a state of impaired immunity follows the initial hyperinflammatory phase of sepsis (8, 10, 27, 32). An important feature of immunosuppression is T cell exhaustion, which was recently recognized following many trials for sepsis involving immunoregulatory therapies, such as PD-1 blockade, interleukin 7 administration, interleukin 15 administration, IFN-γ administration, and CTLA-4 blockade (2, 21, 31, 33). PD-1 is considered to be one of the most promising targets for immunomodulatory therapy in sepsis (31, 33–35), and further studies have confirmed that anti-PD-1 treatment did not meet expectations in all conditions because multiple negative costimulatory molecules are expressed on the surface of exhausted T cells. Researchers have also passively explored multitarget combination blockades, such as combining anti-PD-1 and anti-CTLA-4 therapies, to maximize the recovery of T cells and obtain better therapeutic effects (31, 34). In fact, our understanding of the roles and mechanisms of these negative costimulatory molecules in sepsis is rather limited. Recently, the potential relationship between PD-1 and LAG3 was reported in other non-septic diseases (12, 15, 17, 18), and moreover, LAG3 activation can resist the therapeutic effect of PD-1 blockade (16), but their role in sepsis is still unclear.

Due to the special pathophysiological status of critically ill patients, such as stress state, adrenal secretion level, nutrient intake, and so on, were differently from those of healthy volunteers. In addition, there has been much research on comparing the sepsis with healthy volunteers (5, 8, 10, 23, 31). In particular, it needs to be emphasized, the expression of LAG3 and PD-1 on the surface of T cells of healthy volunteers will not change significantly with time goes on (23, 31). In order to reduce baseline differences in immune status, as much as possible, between critically ill patients and controls, and to highlight the expression changes of negative costimulatory molecules in T cells of septic patients, we designed this study to compare non-septic and septic critically ill patients without comparing with healthy volunteers. It should be noted that this pairwise group study was only used to compare the dynamic changes of negative costimulatory molecules, and did not involve functional analysis of T cells with different phenotypes and their clinical prognostic effects. Therefore, this study does not need to compare with the healthy control group, and its conclusions are not biased. As the control subjects, the non-septic population was selected to be approximately age and sex matched with the septic population. There was no significant difference in the degree of critical illness between the two groups, either in the SOFA scores (*P* > 0.05) or in the APACHE II scores (*P* > 0.05). More importantly, we used strict exclusion criteria; all other known diseases and a history of medication that could affect host immunity were excluded. We finally enrolled 26 subjects and 18 controls from 676 candidates, and many of those excluded patients met the sepsis criteria, might be included in previous other sepsis studies, but those patients themselves more or less combined with immune damage factors and could not really reflect T-cell function. Although the inclusion of patients was relatively narrow and the general representation might be even lost, these simple patients with non-immunocompromised comorbidities were more able to reflect the expression changes of negative costimulatory molecules LAG3 and PD-1 in the case of sepsis, as well as the clinical prognosis changes brought by such changes. With this preliminary study, we can proceed to the related study of broad standard enrollment. As for the identification of sepsis on the 1st day, we made a daily diagnostic evaluation for the infected patients highly suspected for sepsis, using sepsis 3.0 criteria. In this study, we found that PD-1 and LAG3 have unique expression characteristics in sepsis. Although LAG3 plays an important role in T cell inhibition in other diseases, such as cancer (36–39), autoimmune diseases (40–42), chronic viral infections (43, 44), and parasites (13–15, 45), its role in sepsis is not well-understood. Given the changes in LAG3 and PD-1, we need to closely examine their specific roles in sepsis.

The function test confirmed that T cells with double-positive expression of LAG3 and PD-1 were significantly depleted, while T cells with single-positive expression of LAG3 or PD-1 still had certain secretory and proliferative capacities (Figure 6). Moreover, in most patients, the secretory function of the T cells with double-positive expression of LAG3 and PD-1 was twice as damaged as that of the T cells with any single-positive expression pattern. Similar results were also obtained by comparing the apoptosis rate and proliferative ability of the T cells with double-positive expression of LAG3 and PD-1 with those of any single-positive T cells (Figures 7, 8). T cells with double-positive expression showed extremely weak proliferative ability, and more than 80% of the cells remained in the parental cell state (Figure 8B). Even if proliferation occurred, there were not many generations, with more cells in generation 3 or so; in contrast, the control group had more cells in the seventh generation and beyond. Likewise, T cells with single-positive expression of LAG3 or PD-1 still retain a certain proliferative capacity. There were many reports about the exhaustion of T cells caused by the increase of PD-1 expression (46–51), and a decrease in the function of T cells with single positive PD-1 expression also could be found in this acute sepsis study, but not significant exhausted. Maybe there are some other mechanisms that contribute to T cell exhaustion in chronic viral infections. In sepsis patients, the proportion of double-positive CD8^+^ T cells was significantly higher than that of CD4^+^ T cells, suggesting that the synergistic inhibitory effect of the two was more prominent on CD8^+^ T cells, although the proportion of double-positive CD4^+^ T cells was also significantly higher than that of the control group (Figure 5). Because sepsis is generally associated with an absolute decrease in the lymphocyte count, we further analyzed the relationship between the proportion of T cells with double-positive expression of LAG3 and PD-1 and the absolute number of lymphocytes and found that there was a strong negative correlation between these parameters. This finding also confirmed the pathological nature of this relationship, as the proliferative capacity of LAG3 and PD-1 double-positive T cells was weakened while the apoptosis rate was increased. However, this relationship was not found between the absolute lymphocyte count and T cells with single-positive expression of PD-1 or LAG3 in this study.

Furthermore, we analyzed the relationships between the proportion of LAG3 and PD-1 double-positive T cells and relevant clinical indicators. Interestingly, the double-positive proportion was positively correlated with the degree of sequential organ injury (SOFA score) (Figure 9A). Further analysis showed that the higher the proportion of T cells with double-positive expression of LAG3 and PD-1 was, the more serious the organ damage, the longer the hospital stay, the higher the mortality, and the lower the survival rate (Figure 9C). In this study, the proportion of double-positive T cells was always below 25%, while the proportion of double-positive T cells in the control group was below 5%, and no septic patients died when the proportion of double-positive T cells was <5%. That is why we set 5% as a cut-off. Due to the potential synergistic effect of PD-1 and LAG3 and its significant influence on clinical prognostic indices, the therapeutic strategies for immunomodulatory therapy may need to be adjusted in the future. A previous study showed that delaying the use of PD-1 blockade to after 24 h of sepsis could improve the survival of mice with sepsis to some extent (52). When that result is combined with our finding of the unique expression features of PD-1, it seems that the expression of PD-1 in acute sepsis is more likely to be passively increased to prevent the uncontrolled inflammatory cascade and the late coexpression of LAG3 may be the key to T cell exhaustion. Therefore, the use of anti-PD-1 treatment in too early or too late periods will not produce satisfactory therapeutic effects. When used too early, the cascade of inflammatory responses can get out of control, resulting in more early death. When used in too late stages, the activation of LAG3 will certainly affect the effect of the anti-PD-1 therapy, not only in sepsis but also in cancer (16).

Although we systematically analyzed the expression characteristics and functional relationship between LAG3 and PD-1 in T cells from septic patients, and obtained some important results, which may lead to a change in the strategy of immunomodulatory therapy for sepsis in the future, there is still a small limitation. Due to the strict exclusion criteria, although we had many candidates, the number of patients actually included in the study was small. In addition, although we revealed a potential synergistic role for LAG3 and PD-1 in mediating the progressive depletion of T cells, this study, like other studies (12, 18, 19, 53), was not been able to elucidate the mechanism of this synergistic effect because the downstream signaling pathway of LAG3 is poorly understood at present (13). However, we believe that future researchers will be able to shed light on the synergistic mechanisms between LAG3 and PD-1, and multicenter, larger sample clinical studies are expected to confirm the significance of this synergy to help improve the clinical management and prognosis of patients as early as possible.

## Data Availability

The data that support the findings of this study are available from the corresponding author upon reasonable request.

## Ethics Statement

This study was approved by our local ethical review committee in compliance with the declaration of Helsinki. Written and informed consent was obtained from all patients enrolled. (Ethics Committee of the First Affiliated Hospital of Chongqing Medical University, Chongqing, China, the ethical document NO. 2018-017).

## Author Contributions

BN and GR designed the experiments. BN, HD, YS, YX, and ZY performed the experiments and analyzed the data. FZ, YS, LW, and YW collected and analyzed the clinical data. GR further polished the manuscript. GR and HD authorized the publication of the manuscript.

### Conflict of Interest Statement

The authors declare that the research was conducted in the absence of any commercial or financial relationships that could be construed as a potential conflict of interest.

## Abbreviations

PD-1: Programmed cell death 1
LAG3: Lymphocyte-activation gene 3
AECOPD: Acute Exacerbation of Chronic Obstructive Pulmonary Disease
ICU: Intensive Care Unit
SOFA: Sequential [Sepsis-related] Organ Failure Assessment
APACHE II: Acute Physiology and Chronic Health Evaluation II
HIV: Human Immunodeficiency Virus
RPMI: Roswell Park Memorial Institute
EDTA-2Na: disodium ethylenediaminetetraacetate dihydrate
FBS: Fetal Bovine Serum
BSA: Bull Serum Albumin
MESF: Molecules of Equivalent Soluble Fluorochrome
PBS: Phosphate Buffer Saline
FSC: Forward Scatter
SSC: Side Scatter
ELISA: Enzyme-linked immuno sorbent assay
IL-2: human interleukin-2
IL-6: human interleukin-6
TNF-α: tumor necrosis factor-alpha
IFN-γ: interferon-gamma.
